# A novel stroke rehabilitation strategy and underlying stress granule regulations through inhibition of NLRP3 inflammasome activation

**DOI:** 10.1111/cns.14405

**Published:** 2023-08-15

**Authors:** Qingzhu Wang, Wesley Kohls, Melissa Wills, Fengwu Li, Qi Pang, Xiaokun Geng, Yuchuan Ding

**Affiliations:** ^1^ China‐America Institute of Neuroscience Beijing Luhe Hospital, Capital Medical University Beijing China; ^2^ Department of Neurosurgery Wayne State University School of Medicine Detroit Michigan USA; ^3^ Department of Neurosurgery, Shandong Provincial Hospital Shandong University Jinan China; ^4^ Department of Neurology, Beijing Luhe Hospital Capital Medical University Beijing China

**Keywords:** DEAD‐box RNA helicase 3X (DDX3X), ischemic stroke, neuroplasticity, NLRP3 inflammasome, stress granules

## Abstract

**Objective:**

Dynamic changes in ischemic pathology after stroke suggested a “critical window” of enhanced neuroplasticity immediately after stroke onset. Although physical exercise has long been considered a promising strategy of stroke rehabilitation, very early physical exercise may exacerbate brain injury. Since remote ischemic conditioning (RIC) promotes neuroprotection and neuroplasticity, the present study combined RIC with sequential exercise to establish a new rehabilitation strategy for a better rehabilitative outcome.

**Methods:**

A total of 120 adult male Sprague‐Dawley rats were used and divided into five groups: (1) sham, (2) stroke, (3) stroke with exercise, (4) stroke with RIC, and (5) stroke with RIC followed by exercise. Brain damage was evaluated by infarct volume, neurological deficit, cell death, and lactate dehydrogenase (LDH) activity. Long‐term functional outcomes were determined by grid walk tests, rotarod tests, beam balance tests, forelimb placing tests, and the Morris water maze. Neuroplasticity was evaluated through measurements of both mRNA and protein levels of synaptogenesis (synaptophysin [SYN], post‐synaptic density protein‐95 [PSD‐95], and brain‐derived neurotrophic factor [BDNF]) and angiogenesis (vascular endothelial growth factor [VEGF], angiopoietin‐1 [Ang‐1], and angiopoietin‐2 [Ang‐2]). Inflammasome activation was measured by concentrations of interleukin‐18 (IL‐18) and IL‐1β detected by enzyme‐linked immunosorbent assay (ELISA) kits, mRNA expressions of NLR pyrin domain containing 3 (NLRP3), apoptosis‐associated speck‐like protein containing a C‐terminal caspase recruitment domain (ASC), IL‐18 and IL‐1β, and protein quantities of NLRP3, ASC, cleaved‐caspase‐1, gasdermin D‐N (GSDMD‐N), and IL‐18 and IL‐1β. Stress granules (SGs), including GTPase‐activating protein‐binding protein 1 (G3BP1), T cell‐restricted intracellular antigen‐1 (TIA1), and DEAD‐box RNA helicase 3X (DDX3X) were evaluated at mRNA and protein levels. The interactions between DDX3X with NLRP3 or G3BP1 were determined by immunofluorescence and co‐immunoprecipitation.

**Results:**

Early RIC decreased infarct volumes, neurological deficits, cell death, and LDH activity at post‐stroke Day 3 (*p <* 0.05). All treatment groups showed significant improvement in functional outcomes, including sensory, motor, and cognitive functions. RIC and exercise, as compared to RIC or physical exercise alone, had improved functional outcomes after stroke (*p <* 0.05), as well as synaptogenesis and angiogenesis (*p <* 0.05). RIC significantly reduced mRNA and protein expressions of NLRP3 (*p <* 0.05). SGs formation peaked at 0 h after ischemia, then progressively decreased until 24 h postreperfusion, which was reversed by RIC (*p <* 0.05). The assembly of SGs consumed DDX3X and then inhibited NLRP3 inflammasome activation.

**Conclusions:**

RIC followed by exercise induced a better rehabilitation in ischemic rats, while early RIC alleviated ischemia‐reperfusion injury via stress‐granule‐mediated inhibition of NLRP3 inflammasome.

## Graphical abstract

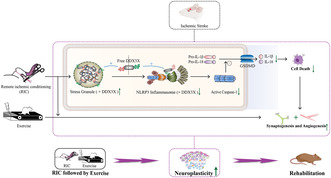

## 1 INTRODUCTION

Although the stroke survival rate has greatly improved due to advances in medical technology and interventions such as thrombolysis, many stroke survivors remain disabled and require long‐term post‐stroke care. Therefore, rehabilitation is an important treatment for stroke patients.^1^, ^2^ In all guidelines, initiation of rehabilitation as soon as possible is recommended to maximize its effectiveness, as the benefits decrease with time.^3^, ^4^ Neuroplasticity is active in the early post‐stroke stage (within 24 h), which is enhanced by early rehabilitation therapy,^5^, ^6^ leading to post‐stroke functional recovery. Therefore, an early rehabilitation strategy is imperative for stroke patients. However, the acute stage of stroke is a sensitive period for neural damage^7^, ^8^, ^9^ and very early exercise may aggravate brain damage.^10^, ^11^, ^12^, ^13^ A new rehabilitation strategy needs urgent development that supplements the deficiencies of the current rehabilitative methods.

Remote (limb) ischemic conditioning (RIC) has been recognized as a method for neuroprotection and neuroplasticity.^14^, ^15^, ^16^ RIC is safe and feasible in stroke patients during the acute stage, when neurons around the infarction are vulnerable and sensitive to injury after stroke,^7^, ^8^ but it has not been thoroughly studied in stroke rehabilitation.^17^, ^18^, ^19^ Thus, we choose RIC initiated at 6 h after reperfusion for neuroprotection that reduces brain injury and thus optimizes the tissue environment for further neuroplasticity and neurorehabilitation induced by the following exercise.^15^, ^20^, ^21^, ^22^ Additionally, exercise would improve balance and coordination, cardiorespiratory fitness, muscle strength, and endurance in stroke patients compared with RIC.^3^, ^23^, ^24^ Taken together, we proposed a novel model of post‐stroke rehabilitation, which combined RIC with sequential exercise for better neurorehabilitation.^25^ Studies have demonstrated that exercise or RIC decreased brain injury, and promoted synaptogenesis and angiogenesis.^15^, ^26^, ^27^, ^28^ NLRP3 inflammasome activation was reported to be a vital mediator of brain injury after ischemic stroke, which could trigger an inflammatory response, induce neuronal cell death, and aggravate brain injury.^29^, ^30^, ^31^ A previous study demonstrated that NLRP3 inflammasome formation was precluded by the induction of stress granules (SGs).^32^ SGs are non‐membrane aggregates of proteins and mRNAs, produced in the cytoplasm under various harmful environmental stimuli to protect valuable mRNAs and proteins from injury.^33^ It has been suggested that the formation of SGs leads to the sequestration of DEAD‐box RNA helicase 3X (DDX3X).^34^ DDX3X is a DEAD‐box RNA helicase that has diverse biological effects.^35^ It also interacts with NLRP3 to regulate the assembly of the NLRP3 inflammasome.^33^ In the present study, we determined that early RIC alleviated ischemia‐reperfusion injury via stress‐granule‐mediated inhibition of the NLRP3 inflammasome and that neurorehabilitation was strengthened with subsequent exercise after RIC. In the present study, we determined that early RIC alleviated ischemia‐reperfusion injury via stress‐granule‐mediated inhibition of the NLRP3 inflammasome and that neurorehabilitation was strengthened with subsequent exercise after RIC. The present study was an extension to a prior study, in which we preliminarily established a novel early stroke rehabilitation concept, the RIC followed by exercise,^25^ and hypothesized that our new protocol with a later initiation of exercise may improve stroke rehabilitation outcomes.

## 2 MATERIALS AND METHODS

### 2.1 Animals

**FIGURE 1:**
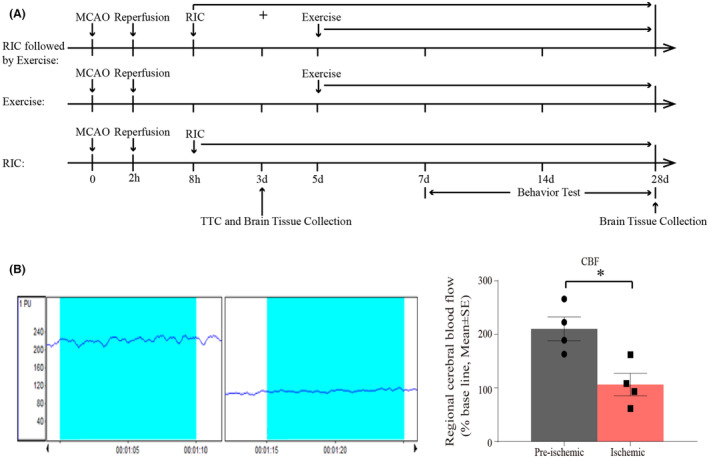
(A) Illustration of the experimental timelines. (B) Representative images and quantification of CBF monitoring of the rats for 2 min before and after the onset of ischemia.

A total of 120 adult male Sprague‐Dawley rats (280–300 g, Vital River Laboratory Animal Technology Co., Ltd.) were used in this study. The protocol was approved by the Animal Care and Use Committee of the Capital Medical University, and the study was conducted in accordance with the National Institutes of Health Guide for the Care and Use of Laboratory Animals (USA). The animals were randomly divided into five groups: (1) sham (*n* = 16), (2) stroke (*n* = 56), (3) stroke with exercise (*n* = 8), (4) stroke with RIC (initiated at 6 h after reperfusion for 3 or 28 days) (*n* = 32), and (5) stroke with RIC followed by exercise (initiated with RIC at 6 h after reperfusion followed by exercise at Day 5 and up to Day 28) (*n* = 8). Rats in the stroke group were sacrificed at 0, 6, 24 h, Day 3, and Day 28 after reperfusion for morphological and molecular analysis. Additionally, rats in the other groups were sacrificed at 3 and 28 days after reperfusion for morphological and molecular analysis. Eight rats from each group were used. After tissue collection, the 50 mg infarcted ipsilateral brain tissue was taken and the lysate was added in proportion to sample weight: tissue protein lysate volume 1:10 for immunoblot analysis, 10 mg for enzyme‐linked immunosorbent assay (ELISA) assay, and 50 mg for co‐immunoprecipitation (co‐IP) assay. Then, 15 mg tissue samples were taken and the 300 μL trizol reagent was added for the PCR analysis. The experimental timeline is shown in Figure 1A. The outcome assessment was blindly performed.

### 2.2 Focal cerebral ischemia

Briefly, rats were initially anesthetized in a chamber with 3% isoflurane and a mixture of 70% nitrous oxide and 30% oxygen, which was maintained with a facemask using 1% isoflurane delivered from a calibrated precision vaporizer.^36^ Poly‐L‐lysine‐coated intraluminal nylon (4.0) sutures were used to yield consistent infarcts, greatly reducing inter‐animal variability. The external carotid artery (ECA) of the rat was tied, and Poly‐L‐lysine‐coated intraluminal nylon (4.0) sutures were inserted from the common carotid artery (CCA) to the internal carotid artery (ICA) via ECA. The nylon sutures were then used to block the MCA at its origin (18 mm). After ischemia for 2 h, the plug was removed for reperfusion. During the unilateral, 2‐h middle cerebral artery occlusion (MCAO), cerebral blood flow (CBF), blood pCO_2_ and pO_2_, mean arterial pressure, and rectal temperature were continuously monitored. Rectal temperatures were maintained between 36.5 and 37.5°C using a circulating heating pad and a heating lamp. PeriFlux System 5000 was used to measure CBF of the rats for 2 min before and after the onset of ischemia. Regional CBF was significantly decreased at the onset of MCAO (Figure 1B). The modified scoring systems (Zea Longa [5 scores] and Belayev et al. [12 scores]) were used to determine neurological deficits.^36^ The ischemic rats with scores of 2 or below or dead (about 10%), showing an unsuccessful surgery, were excluded for further analyses.

### 2.3 Treadmill exercise

Initiated at Day 5, ischemic rats were run on a four‐lane treadmill (ZS‐PT‐II, ZS Dichuang Instruments, Inc.) at 5 m/min for the first 10 min, 9 m/min for the second 10 min, and 12 m/min for the last 10 min on days 1 and 2, and then 12 m/min for 30 min on the third and subsequent days, as previously described by us.^26^ All animals were housed in groups of three in standard cages for equal times.

### 2.4 RIC

RIC was initiated at 6 h of reperfusion (8 h of MCAO). RIC was conducted daily for 3 cycles. The cycles of ischemia (10 min) and reperfusion (10 min) were performed by placing and releasing a thin elastic tourniquet firmly around the upper third of the hind limb at the level of the femoral arteries, as previously described by us.^37^ The limb ischemia was confirmed by limb cyanosis within 20 s while the reperfusion was confirmed as the skin color returned to its normal pink shade within 20 s.

### 2.5 RIC followed by exercise

The RIC and exercise regimen was conducted as follows: (1) RIC was initiated at 6 h of reperfusion up to Day 28 and (2) exercise was followed at Day 5 of reperfusion and continued until Day 28. This experimental timeline was shown in Figure 1A. Exercise was initiated when spontaneous penumbra recovery reached its peak (Day 5 after stroke) according to the post‐stroke dynamic pathologic features.^7^ The specific methods of the corresponding RIC and treadmill exercise were the same as above.

### 2.6 Neurological deficits

The modified scoring systems (5 and 12 scores) proposed by Zea Longa (5 scores) and Belayev et al. (12 scores) were used to examine the severity of neurological deficits in rats before and after 24 h of reperfusion.^36^ The severity and consistency of brain damage within each group was highly important in this study. After MCAO, the modified scoring systems for neurological deficits were used to confirm brain injury, and rats with scores of 2 or below or dead were considered to represent the unsuccessful establishment of the MCAO model and were consequently excluded (about 10%). Exclusion was then confirmed on autopsy by lack of an ischemic core, indicating an unsuccessful surgery.

### 2.7 Neurobehavioral tests

These tests included adhesive removal, beam balance, forelimb placing, grid walking, and rotarod performance (R03–1; Xin‐Ruan Instruments, Inc.), as assessed at days 7, 14, and 28. The Morris water maze (ZS‐II; ZS Dichuang Instruments, Inc.) was also employed, at 23–28 days; this is following a previous report that showed no obvious suppressive effect on swimming at 23 days after the ischemic event.^38^ In the grid walk test, rats were placed on a wire grid (100 × 25 × 50 cm) and allowed to walk from one end to the other; the total number of foot slips during this crossing was recorded. In the rotarod test, rats were placed on a rotating drum that accelerated from 4 to 40 rpm within 300 s; the time that the animals stayed on the rotating rod was then recorded. In the beam balance test, rats were placed on a narrow wooden beam (122 × 2.5 × 42 cm), and performance was scored from 0 to 6 (0 = no attempt to stay on the beam; 1 = attempted to stay on the beam but no movement; 2 = attempted to cross the beam but failed; 3 = crossed the beam with contralateral hindlimb slips >50% of the time; 5 = crossed the beam with contralateral hindlimb slips <50% of the time; 6 = crossed the beam without slips).^38^ In the forelimb placing test, rats were held gently with forelimbs close to the tabletop, while the surface was lightly brushed using each side of their vibrissa. The ability of rats to place the preferred forelimb on the edge of the table in this context was recorded 10 times, and then, placing rates were calculated. Finally, in the Morris water maze, rats were placed into a pool (diameter = 150 cm) at one of the four locations and allowed to swim for 90 s to find a hidden platform (diameter = 10 cm); swim speed and the time taken to find the hidden platform were recorded using a camera positioned above the pool that transmitted data to an analysis system for calculation. Tests were conducted on non‐consecutive days to mitigate possible confounding due to motor learning that might have occurred if these tests were performed in close succession.

### 2.8 Cerebral infarct volume

After 3 days of reperfusion in ischemic rats, their brains were resected and cut into 2‐mm‐thick slices (brain matrix) and treated with TTC (Sigma‐Aldrich) for staining.^12^ An indirect method for calculating infarct volume was used to minimize error caused by edema.

### 2.9 TUNEL assay

The TUNEL assay was used to investigate DNA fragmentation, which was performed with a commercial kit (In situ Cell Death Detection Kit, Fluoresce, Roche) as outlined in our previous research.^39^ In accordance with the manufacturer's instructions, the fixed slides were washed three times for 5 min with PBS and permeabilized with 0.1% (v/v) Triton X‐100 containing 0.1% (w/v) sodium citrate for 2 min on ice. Then, the slides were incubated in 50 μL of the TUNEL reaction mixture at 37°C for 1 h. Slides were washed three times with PBS. The positive TUNEL staining was visualized using a fluorescence microscope (DM4000, Leica). Images were randomly acquired from different positions of the infarcted cortex. The TUNEL^+^ cells were then counted with the manual cell counting tool in ImageJ. Values from all sections in each group were obtained in a blinded manner and used for subsequent statistical analysis. The percentage of TUNEL+ positive cells (%) = (TUNEL^+^ cells in all 16 regions/total cells in all 16 regions) × 100%.

### 2.10 ELISA

As previously described by us,^39^ 500 μL of extraction lysis buffer was added to the tube for every 10 mg of infarcted tissue and then mixed with an electric homogenizer. ELISA kits were used to measure cell death (m1059429, Shanghai Enzyme‐linked Biotechnology Co.), lactate dehydrogenase (LDH) (11,644,793,001, Roche), interleukin‐18 (IL‐18) (ml002816, Shanghai Enzyme‐linked Biotechnology Co.), and IL‐1β (ml037361, Shanghai Enzyme‐linked Biotechnology Co.).

### 2.11 Expression of mRNA by real‐time PCR

After all experimental procedures, isolated cerebral samples were homogenized, isolating RNA using the trizol reagent (Invitrogen) as previously described by us.^40^ Total RNA was then converted into cDNA using the High Capacity cDNA Reverse Transcription Kit (Applied Biosystems). The quantification of gene expression was determined by Prism 7500 real‐time PCR (Applied Biosystems). All reactions were performed under the following conditions: 95°C for 15 min, 40 cycles of 95°C for 10 s, and 60°C for 30 s. β‐actin was used as the control gene, and all data are represented as relative mRNA expression on gene expression. The primers for rat NLRP3, C‐terminal caspase recruitment domain (ASC), IL‐1β, IL‐18, GTPase‐activating protein‐binding protein 1 (G3BP1), T cell‐restricted intracellular antigen‐1 (TIA1), DDX3X, synaptophysin (SYN), post‐synaptic density protein‐95 (PSD‐95), and brain‐derived neurotrophic factor (BDNF), vascular endothelial growth factor (VEGF), angiopoietin‐1 (Ang‐1), and angiopoietin‐2 (Ang‐2), and β‐actin are shown in Table 1.

**TABLE 1:** Primer sequences of each target gene.

Target gene	Forward	Reverse
SYN	5′‐CAGTGGGTCTTTGCCATCTT‐3′	5′‐TTCAGCCGACGAGGAGTAGT‐3′
PSD95	5′‐TGCACTATGCTCGTCCCATCATCA‐3′	5′‐TGTGCCTGGATGTCCTTCTCCATT‐3′
BDNF	5′‐GAGCGTGTGTGACAGTATTAG‐3′	5′‐GTAGTTCGGCATTGCGAGTTC‐3′
Ang‐1	5′‐ATGCGCCCTTATGCTAACAG‐3′	5′‐ TTTAGATTGGAAGGGCCACA‐3′
Ang‐2	5′‐CAAGTGTTCCCAGATGCTCA ‐3′	5′‐ AAGTTGGAAGGACCACATGC‐3′
NLRP3	5′‐CTGCATGCCGTATCTGGTTG‐3′	5′‐GCTGAGCAAGCTAAAGGCTTC‐3′
IL‐18	5′‐ACCGCAGTAATACGGAGCAT‐3′	5′‐TCTGGGATTCGTTGGCTGTT‐3′
IL‐1β	5′‐CAGCTTTCGACAGTGAGGAGA ‐3′	5′‐TTGTCGAGATGCTGCTGTGA ‐3
ASC	5′‐GCACAGCCAGAACAGAACATT‐3′	5′‐CCAGGCTGGAGCAAAGCTAA ‐3′
G3BP1	5′‐CTCAGCCGCACAGGTTGAA ‐3′	5′‐CCTGGTTCAGCAGGGTGTAG‐3′
DDX3X	5′‐GGCCATGAAGATACCTGGTGTTA ‐3′	5′‐CACGGGCCAGCATCTGTATT ‐3′
TIA1	5′‐GGACGAGATGCCCAAGACTC ‐3′	5′‐GTCATTTCCTGCCGTATCCA ‐3
β‐actin	5′‐TCATGAAGTGTGACGTTGACATCCGT‐3′	5′‐CCTAGAAGCATTTGCGGTGCAGGATG‐3′

### 2.12 Protein expression by Western blot

As previously described by us,^40^ proteins were extracted from the MCA‐supplied brain regions and loaded onto SDS‐polyacrylamide gel for electrophoresis. Gel transfer to a PVDF membrane was performed under 200 V for 1 h. Membranes were blocked with 5% skimmed milk. Membranes were incubated with primary antibodies (1:1000, rabbit anti‐NLRP3, Merck; 1:500, rabbit anti‐ASC, Abcam; 1:1000, rabbit anti‐IL‐1β, Abcam; 1:1000, rabbit anti‐IL‐18, Proteintech; 1:1000, rabbit anti‐caspase‐1, Abcam; 1:500, rabbit anti‐GSDMD, Affinity; 1:2000, rabbit anti‐G3BP1, Proteintech; 1:500, rabbit anti‐TIA1, Proteintech; 1:1000, rabbit anti‐DDX3X, Proteintech; 1:1000, rabbit anti‐IgG antibody, Cell Signaling Technology; 1:10,000, rabbit anti‐SYN, Abcam; 1:1000, rabbit anti‐PSD‐95, Cell Signaling Technology; 1:1000, rabbit anti‐BDNF, Abcam; 1:500, rabbit anti‐Ang‐1, Proteintech; 1:500, rabbit anti‐Ang‐2, Proteintech; 1:500, rabbit anti‐VEGF, Abcam; 1:1000, mouse β‐actin, Santa Cruz) for 24 h at 4°C. Next, membranes were incubated with a secondary antibody (1:4000, goat anti‐rabbit IgG, goat anti‐mouse IgG, Cell Signaling Technology) for 2 h at room temperature. Western blot images for each of the antibodies were analyzed using an image analysis program (ImageJ 1.42, National Institutes of Health) to quantify protein expression in terms of relative image density.

### 2.13 Immunofluorescence staining

Immunofluorescence staining was used to detect the colocalization of DDX3X and G3BP1 and the colocalization of DDX3X and NLRP3, as previously described by us.^39^ For immunofluorescence, sections were incubated with primary mouse anti‐DDX3X (Proteintech, 67,915‐1‐Ig) and rabbit anti‐G3BP1 (Proteintech, 13,057‐2‐AP) or rabbit anti‐NLRP3 (Merck, sab5700723) antibodies overnight at 4°C, followed by application of fluorescence‐conjugated secondary antibodies (Alexa Fluor 594 for G3BP1 or NLRP3, 1:500, Thermo Fisher, A‐11005; Alexa Fluor 488 for DDX3X, 1:500, Thermo Fisher, A‐11008) at room temperature for 1 h. An antifade mounting medium with DAPI (Zhongshan Biotechnology) was used to mount the slides. The images were examined with a Leica TCS‐SP5 confocal microscope (Leica).

### 2.14 co‐IP assay

Co‐IP was used to detect the interaction between DDX3X and G3BP1 and between NLRP3 and DDX3X as described previously.^41^ After harvesting tissue samples, total extracts were prepared by brief sonication in a Cell Lysis Buffer (Cell Signaling Technology, 9803). Primary rabbit anti‐G3BP1 (Proteintech, 13,057‐2‐AP), rabbit anti‐DDX3X (Proteintech, 11,115‐1‐AP), rabbit anti‐NLRP3 (Abcam, ab263899), and rabbit anti‐IgG (Cell Signaling Technology, 3900) antibodies were added to 200 μL cell lysate and incubated overnight at 4°C. Lysate and antibody (immunocomplex) solution was transferred to the tube containing protein G magnetic beads (Cell Signaling Technology, 70,024), incubated with rotation for 20 min at room temperature. A Magnetic Separation Rack (Cell Signaling Technology, 7017) was used to separate the magnetic beads according to the manufacturer's instructions and then boiled to denature the protein–bead complex. The immunoprecipitates from cells were subjected to a Western blot analysis.

### 2.15 Statistical analysis

Statistical analyses were performed with SPSS Statistics for Windows, Version 17.0 (SPSS Inc.). The normal distribution test was performed using the Shapiro–Wilk test, and we found the data were normally distributed. Differences among groups were assessed using one‐way ANOVA with a significance level of *p* < 0.05. Comparisons between two distinct groups were considered significant for *p* < 0.05 using unpaired *t*‐test. Post hoc comparison among groups was performed using the least significant difference method. All data were presented as the mean ± standard error of the mean.

## 3 RESULTS

### 3.1 Reduced brain injury by early RIC

**FIGURE 2:**
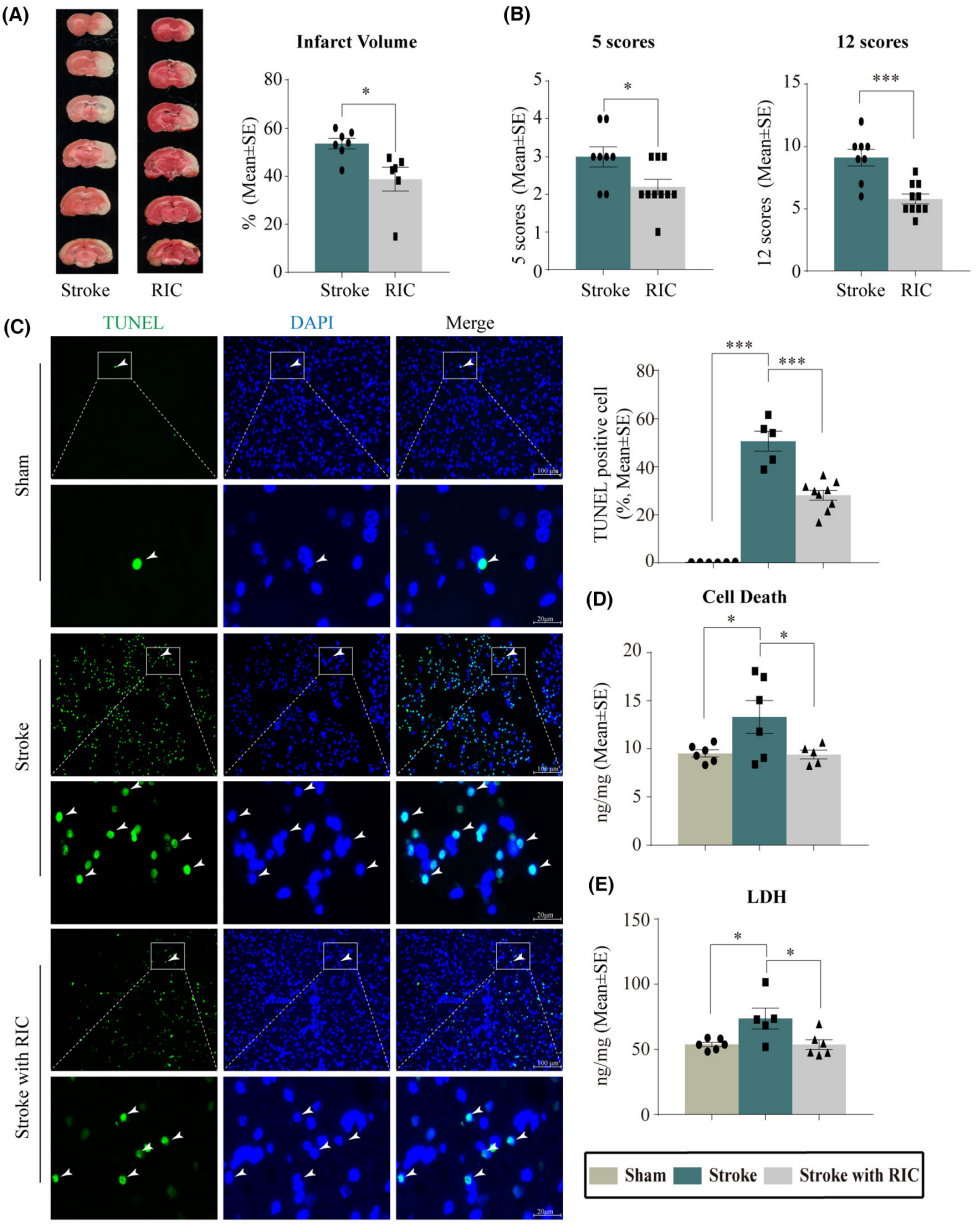
RIC significantly diminished brain injury. (A, B) Assessment of brain damage using TTC staining and neurological deficits evaluation. (A) RIC induced a significant decrease in infarct volume in the penumbra region of the ischemic territory supplied by MCAO at day 3 post reperfusion. The variables between the stroke and RIC groups were analyzed using an unpaired *t*‐test. (B) RIC significantly reduced neurological deficits, as detected by both 5‐ and 12‐point systems. (C) Apoptotic cell death as detected by the TUNEL assay. In the sham group (top two panels), few apoptotic cells were observed (white arrowheads indicating TUNEL+ positive cells). In the stroke group, apoptotic cells were significantly increased (*** *p* < 0.001, middle two panels), which were significantly reduced by RIC (*** *p* < 0.001, bottom two panels). Scale bars represent 100 μm for low and 20 μm for high magnification images. (D, E) Cell death (D) and LDH levels (E) were quantified using ELISA. Data, presented as mean ± SEM. * *p* < 0.05, indicate a significant decrease in both cell death and LDH upregulation as a result of RIC treatment.

TTC histology demonstrated RIC‐induced infarct volume reduction in the penumbra region of the ischemic territory supplied by the middle cerebral artery (Figure 2A). A large infarct (53.6%) was seen after 2 h MCAO followed by 3‐day reperfusion. Early RIC significantly decreased the infarct volumes to 38.8% (*p* < 0.05, Figure 2A). Neurological deficits were decreased significantly in the RIC groups, detected by the 5 or 12 scoring systems after stroke (*p <* 0.05, Figure 2B). In TUNEL assays, as compared to the sham group, cell death was significantly increased after MCAO (*p* < 0.001), while RIC significantly reduced cell death at Day 3 after reperfusion (*p <* 0.001, Figure 2C). Cell death, again determined by ELISA, was elevated by ischemia/reperfusion injury, but markedly decreased by RIC (*p <* 0.05, Figure 2D). Similar trends in LDH level were observed after stroke with RIC (*p <* 0.05, Figure 2E). Together, these results indicated the valuable role of RIC in reducing neural damage after ischemic stroke, suggesting the importance of RIC in our new rehab protocol in the promotion of better rehabilitation efforts.

### 3.2 Improved functional outcomes

**FIGURE 3:**
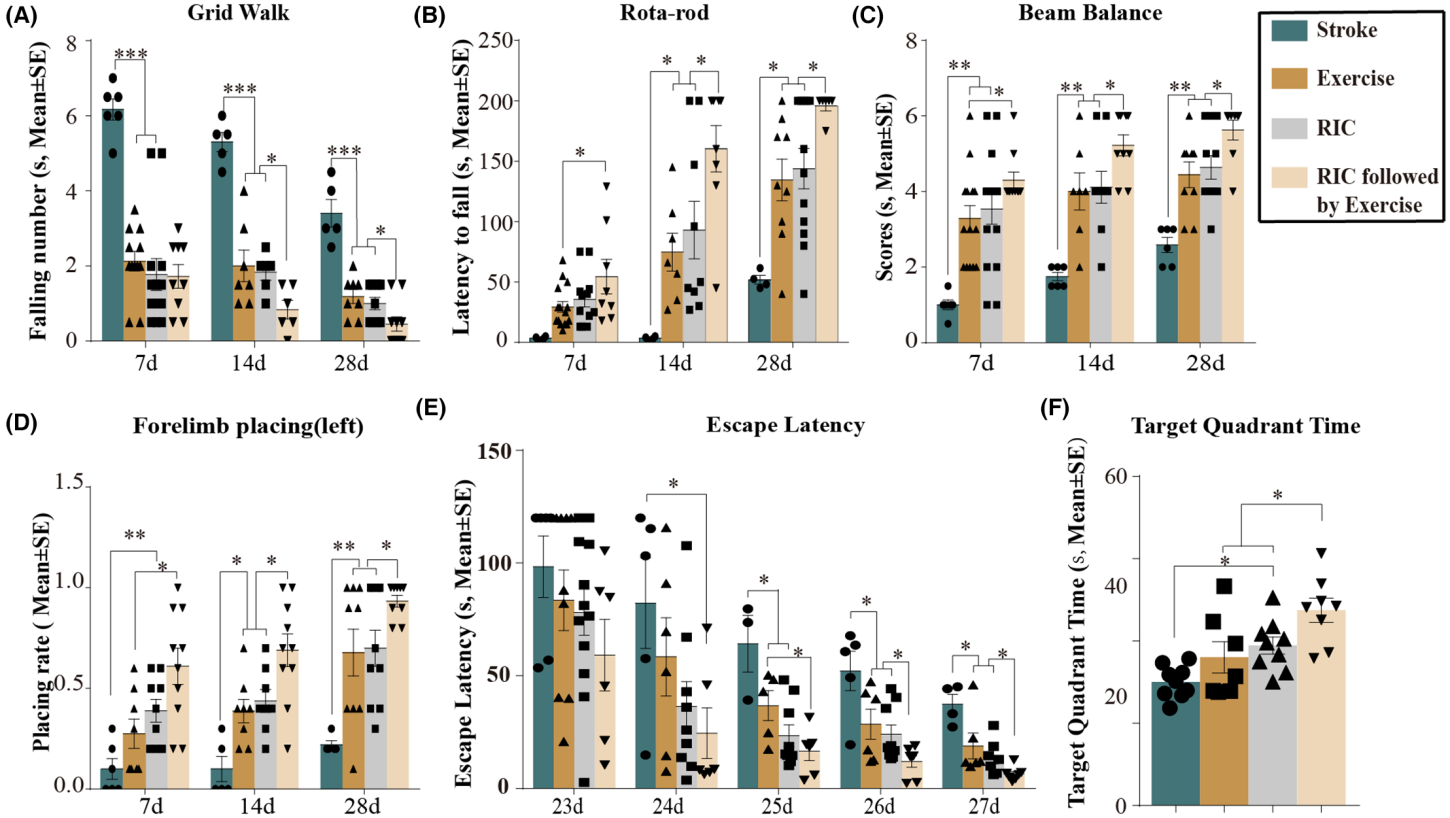
Exercise, RIC, and RIC followed by exercise improved neurobehavioral function. (A) In the grid walk test, the number of foot slips was significantly reduced after exercise, RIC, and RIC followed by exercise as compared to stroke rats without treatment. Furthermore, improvements were seen with RIC followed by exercise. (B) The latency to fall off the rotarod was significantly increased after exercise, RIC, and RIC followed by exercise as compared to stroke rats without treatment. Furthermore, improved performance was seen with RIC followed by exercise. (C) In the beam balance test, the scores were significantly increased after exercise, RIC, and RIC followed by exercise as compared to stroke rats without treatment. Furthermore, improved performance was seen with RIC followed by exercise. (D) In the forelimb placing test, the placing rate was significantly increased after exercise, RIC, and RIC followed by exercise as compared to stroke rats without treatment. Furthermore, improved performance was seen with RIC followed by exercise. (E, F) Learning and memory ability was examined by the Morris water maze test. (E) Latency to locate the submerged platform was significantly shortened after exercise, RIC, and RIC followed by exercise. (F) The rats spent more time in the target quadrant with the hidden platform after exercise, RIC, and RIC followed by exercise. Furthermore, improved performances were seen with RIC followed by exercise. The data were presented as the mean ± SEM. *p < 0.05, **p < 0.01, ***p < 0.001.

In the grid walk test, falling numbers observed in the stroke groups were 6.2 at Day 7, 5.3 at Day 14, and 3.4 at Day 28, which were significantly decreased by all rehabilitation protocols (*p <* 0.001, Figure 3A). Performances of ischemic rats with RIC followed by exercise were further improved on days 14 (*p <* 0.05, respectively) and 28 (*p <* 0.05, respectively) as compared to those with exercise or RIC monotherapy (Figure 3A). Similar trends in neurological function were also seen in all other tests, including rotarod (Figure 3B), beam balance (Figure 3C), and forelimb placing (Figure 3D).

We also evaluated cognitive deficits using the Morris water maze at days 23 through 28 (Figure 3E,F). The two main test metrics for the water maze, escape latency, and time in target quadrant reflected learning and memory, respectively. Exercise, RIC, and our new rehab protocol regimens significantly shortened the latency to locate the hidden platform as compared to the stroked rats at days 23 through 27 (*p <* 0.05, Figure 3E). Performances of ischemic rats with RIC followed by exercise were further improved on days 26 (*p <* 0.05, respectively) and 27 (*p <* 0.05, respectively) as compared to those with exercise or RIC monotherapy (Figure 3E). At Day 28, we removed the platform in the target quadrant to evaluate the memory of rats and we found that ischemic rats without rehabilitation took 27.1 s in the target quadrant while ischemic rats with exercise took 36.0 s, RIC took 38.9 s, and RIC and exercise took 47.4 s. Ischemic rats with rehabilitation regimes spent more time in the target quadrant as compared to the stroke rats without treatment (*p <* 0.05, Figure 3E). Memory of rats with our new rehab strategy was further improved compared with exercise or RIC only (*p <* 0.05, Figure 3F). Together, these results suggested the valuable and superior role of RIC followed by exercise in promoting motor and cognitive functional recovery after ischemic stroke.

### 3.3 Neuroplasticity (Synaptogenesis and Angiogenesis)

**FIGURE 4:**
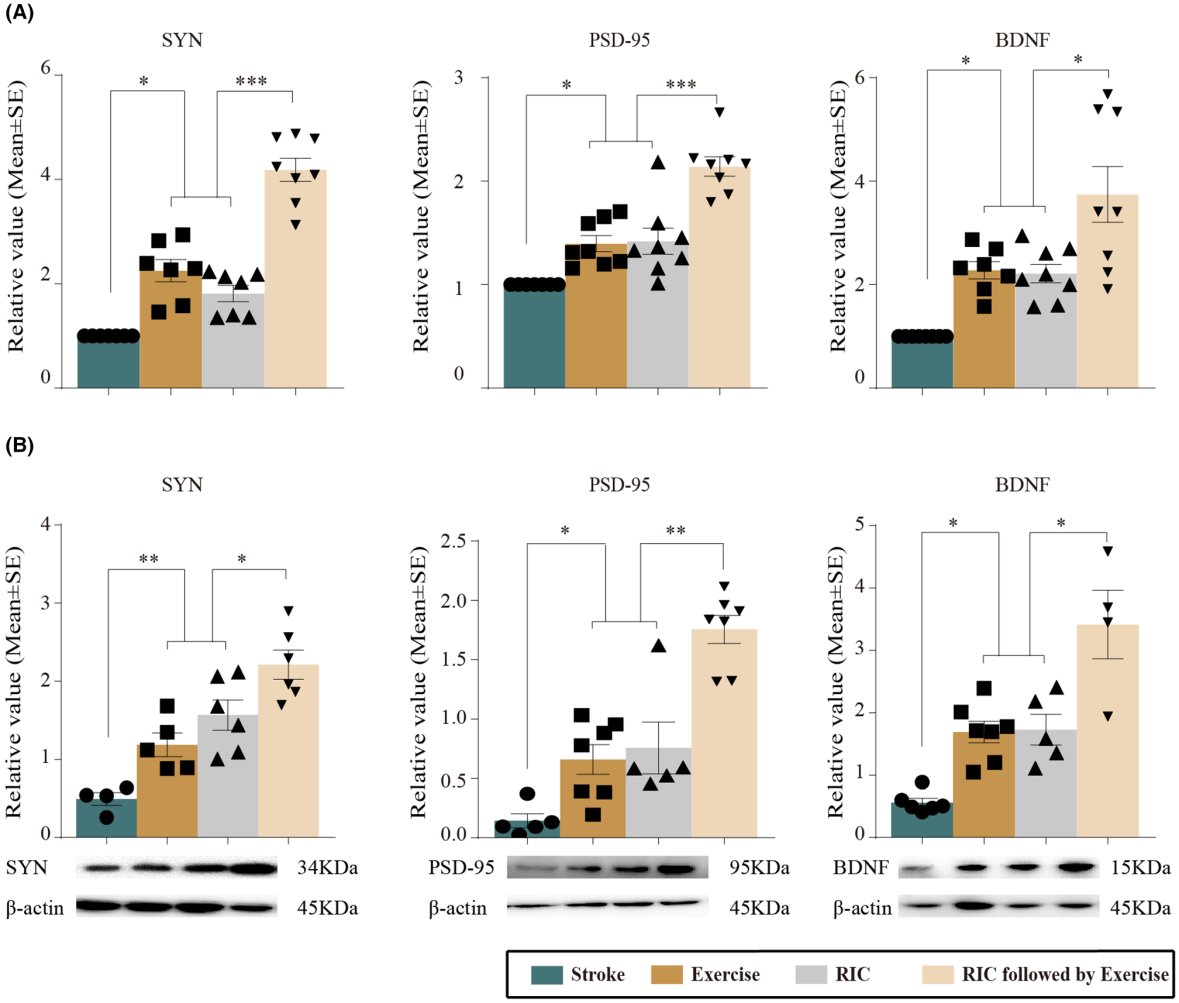
Exercise, RIC, and RIC followed by exercise‐enhanced neuroplasticity and synaptogenesis (SYN, PSD‐95, BDNF), shown by mRNA and protein expressions measured by real‐time PCR and Western blot. (A) mRNA expressions of SYN, PSD‐95, and BDNF were significantly increased after exercise, RIC, and RIC followed by exercise as compared to stroke rats. Furthermore, increases were seen with RIC followed by exercise. (B) Western blot protein measures of SYN, PSD‐95, and BDNF were also significantly increased after exercise, RIC, and RIC followed by exercise in ischemic rats. Furthermore, increases were induced by RIC followed by exercise. The data were presented as the mean ± SEM. *p < 0.05, **p < 0.01, ***p < 0.001.

**FIGURE 5:**
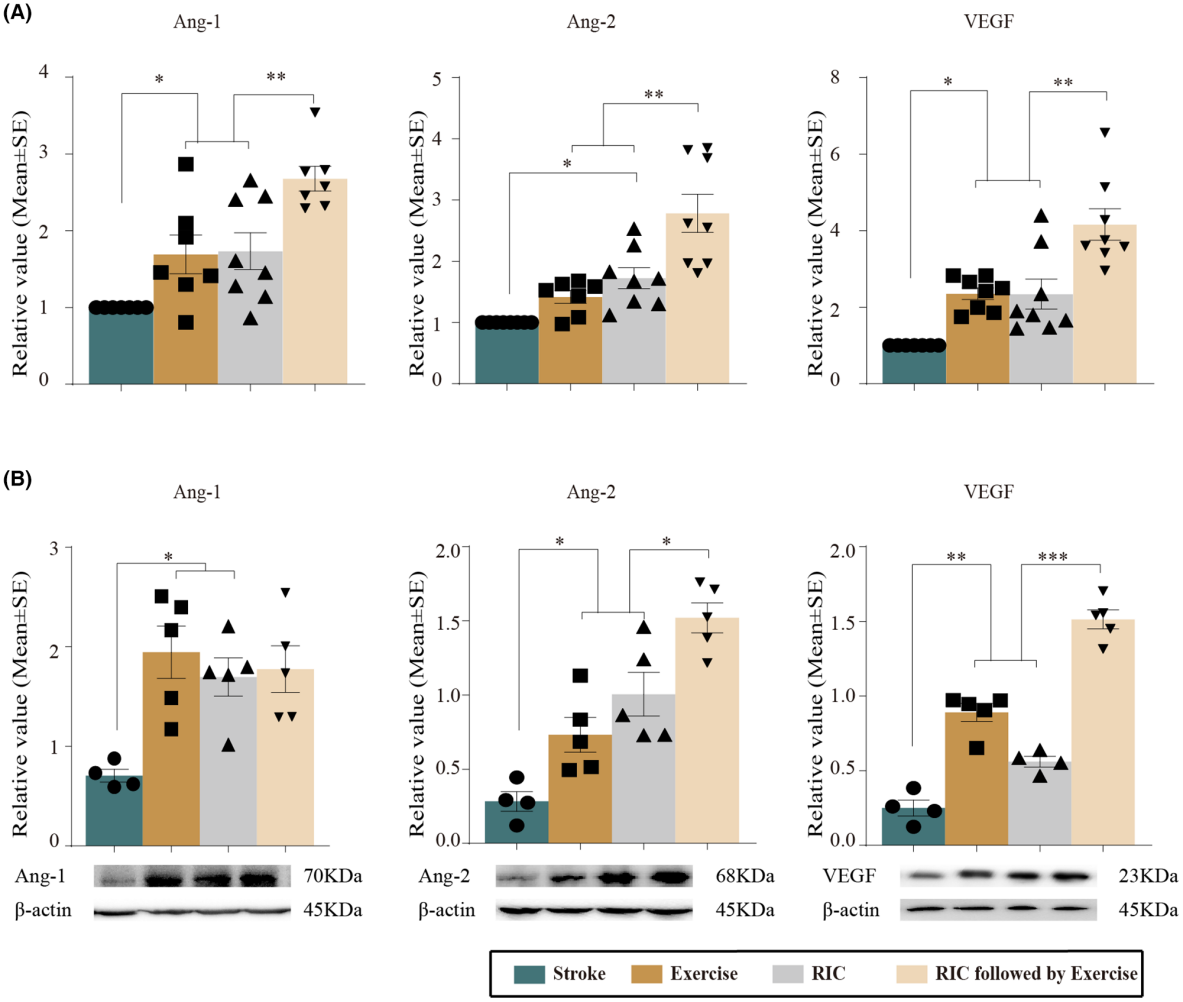
Exercise, RIC, and RIC followed by exercise protocols enhanced angiogenesis, shown by mRNA and protein expressions of Ang‐1, Ang‐2, and VEGF measured by real‐time PCR and Western blot. (A) mRNA expressions of Ang‐1, Ang‐2, and VEGF were significantly increased after exercise, RIC, and RIC followed by exercise as compared to stroke rats. Furthermore, increases were seen with RIC followed by exercise. (B) Protein measures of Ang‐1, Ang‐2, and VEGF were significantly increased after exercise, RIC, and RIC followed by exercise as compared to stroke rats. Furthermore, increases were induced by RIC followed by exercise in Ang‐2 and VEGF. The data were presented as the mean ± SEM. *p < 0.05, **p < 0.01, ***p < 0.001.

Levels of SYN, PSD‐95, and BDNF, which are known to be involved in processes of synaptogenesis, were detected by Western blot (Figure 4). As compared to the stroke without intervention, exercise, RIC, and our new rehab strategy, all significantly increased mRNA and protein expressions of SYN, PSD‐95, and BDNF (*p <* 0.05) at Day 28 of reperfusion. We further found that RIC followed by exercise significantly increased SYN, PSD‐95, and BDNF levels in mRNA and protein expressions as compared to the RIC and exercise monotherapy groups (*p* < 0.05). Exercise, RIC, and our novel rehab protocol all significantly increased expression of angiogenic factors, including Ang‐1, Ang‐2, and VEGF, as compared to the stroke group at Day 28 (*p <* 0.05, Figure 5). Additionally, the mRNA and protein expressions of Ang‐2 and VEGF were enhanced in the RIC followed by exercise group (*p <* 0.05), while Ang‐1 mRNA expression was further increased by the new rehab strategy (*p <* 0.01). Together, these results indicated a significant role of RIC followed by exercise on synaptogenesis and angiogenesis in ischemic rats.

### 3.4 NLRP3 inflammasome activation

**FIGURE 6:**
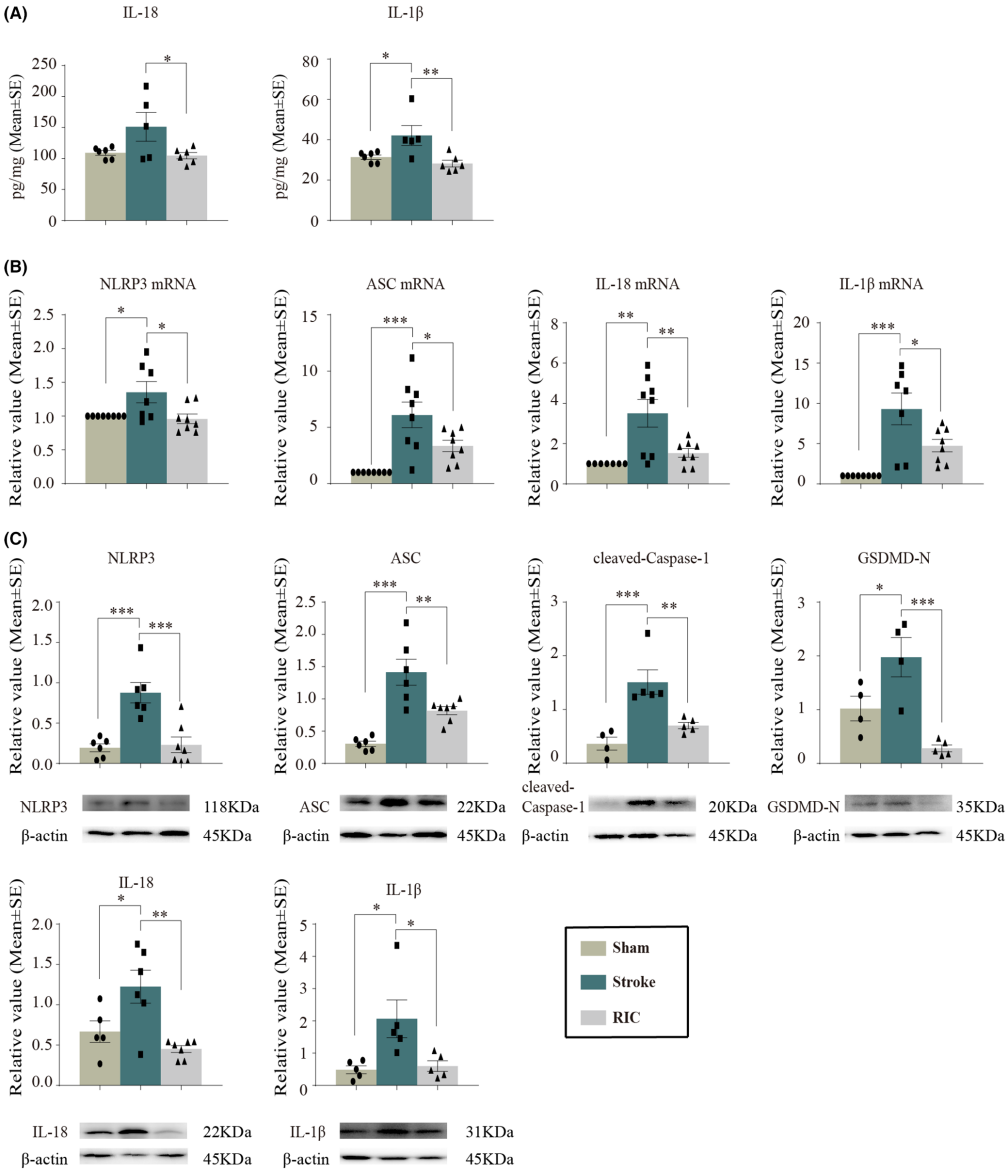
RIC significantly decreased NLRP3 inflammasome activation. (A) RIC decreased IL‐18 and IL‐1β levels after stroke, measured by ELISA. (B) mRNA expressions of NLRP3, ASC, IL‐18, and IL‐1β were significantly decreased after RIC as compared to stroke rats. (C) Western blot protein measures of NLRP3, ASC, cleaved‐caspase‐1, GSDMD‐N, IL‐18, and IL‐1β were also significantly decreased after RIC in ischemic rats. The data were presented as the mean ± SEM. * *p <* 0.05, ** *p <* 0.01, *** *p <* 0.001.

The NLRP3, ASC, GSDMD‐N, IL‐18, and IL‐1β were analyzed at 3 days of reperfusion. Early RIC remarkably ameliorated the notable elevation of IL‐18 and IL‐1β levels after stroke as detected by ELISA (*p <* 0.05, Figure 6A). NLRP3, ASC, IL‐18, and IL‐1β expressions at both mRNA and protein levels were significantly increased after ischemia/reperfusion (*p <* 0.05), while they were decreased by early RIC (*p <* 0.05, Figure 6B,C). Activation of proinflammatory caspase‐1 and their subsequent cleavage of GSDMD resulting in GSDMD N‐terminal fragments were observed to be significantly increased at Day 3 of reperfusion (*p <* 0.05, Figure 6C). Thus, overexpression was significantly reversed by RIC (*p <* 0.01, Figure 6C). Together, these results indicated early RIC inhibited NLRP3 inflammasome activation after ischemic stroke.

### 3.5 Stress granules formation

**FIGURE 7:**
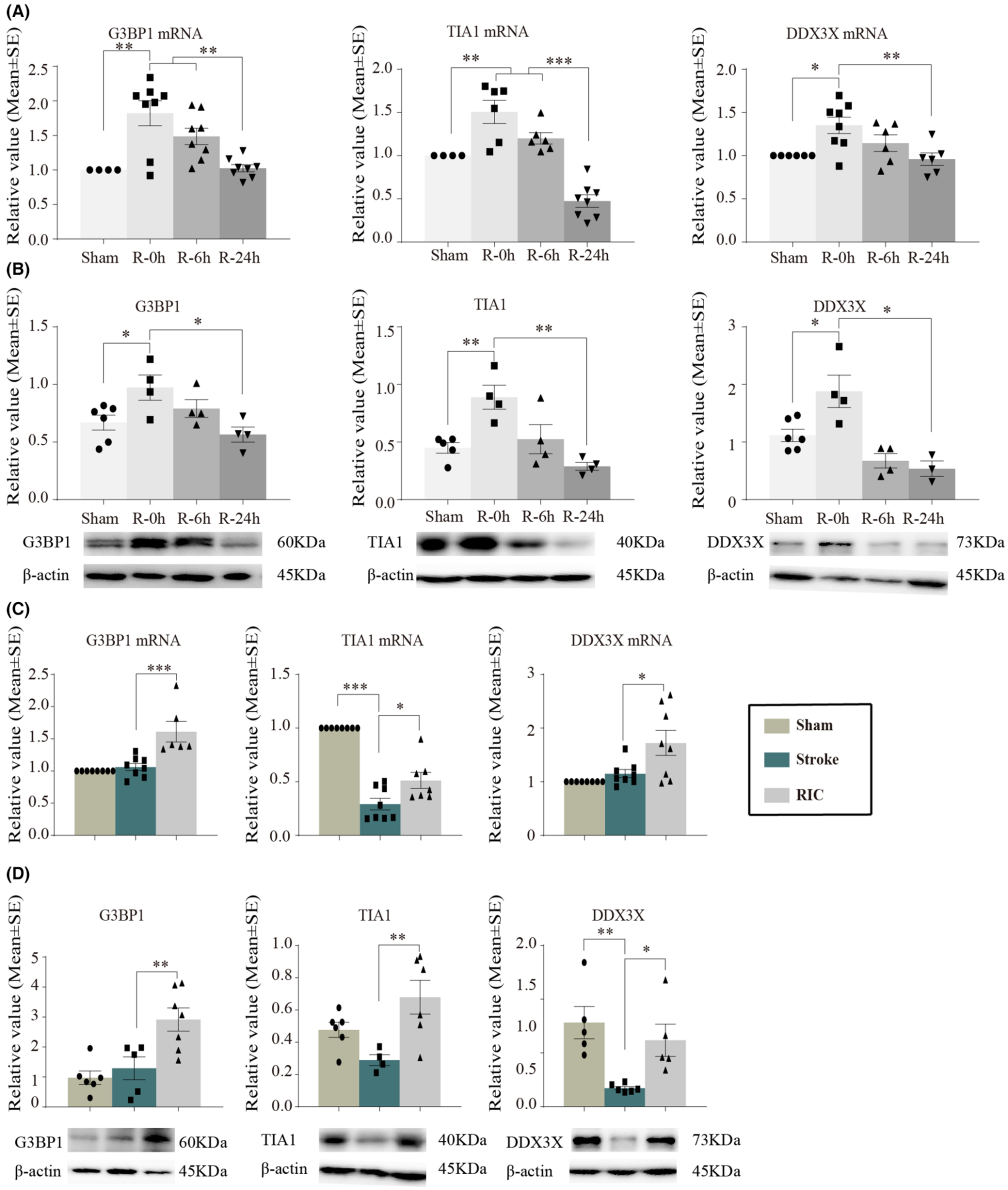
The dynamic generation of SGs at different time points of reperfusion and after RIC in the MCAO model. (A,B) mRNA and protein expressions of G3BP1, TIA1, and DDX3X were significantly increased and then gradually decreased at 0, 6, and 24 h after cerebral ischemia/reperfusion. (C, D) mRNA and protein expressions of G3BP1, TIA1, and DDX3X were significantly increased after RIC in ischemic rats. The data were presented as the mean ± SEM. *p < 0.05, **p < 0.01, ***p < 0.001.

G3BP1, TIA1, and DDX3X were major components of SGs.^42^ The results showed that the mRNA by RT‐qPCR assays and protein expressions of G3BP1 by Western blot analysis were transiently increased at 0 h after cerebral ischemia/reperfusion (R‐0 h) and then was decreased in the R‐24 h groups (*p <* 0.05, Figure 7A,B). A similar trend was observed for the TIA1 and DDX3X expression levels (*p <* 0.05, Figure 7A,B). As presented in Figure 7C,D, RIC significantly increased G3BP1, TIA1, and DDX3X levels in mRNA and protein expressions at Day 3 after stroke. These data suggested SGs formation peaked at 0 h after cerebral ischemia/reperfusion, then progressively decreased until 24 h post‐reperfusion, and increased again due to stimulation by RIC.

### 3.6 The role of DDX3X in SGs formation and the NLRP3 inflammasome activation

**FIGURE 8:**
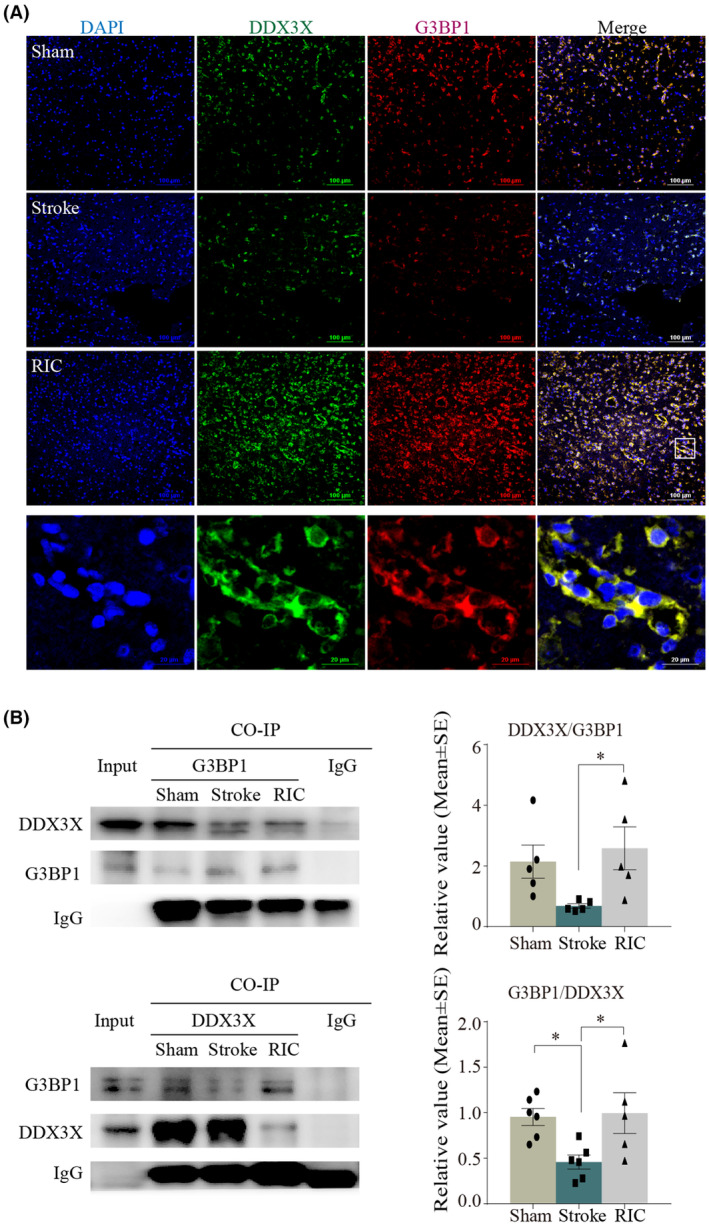
DDX3X was an essential component of SGs in the MCAO model. (A) Representative merged images showing the colocalization of G3BP1 with DDX3X in brain tissue sections. Scale bar = 100 and 20 μm. (Green represented DDX3X; red represented G3BP1; yellow represented colocalization). (B) The co‐IP assay was used to detect the interaction between G3BP1 with DDX3X in the MCAO model. The data were presented as the mean ± SEM. *p < 0.05.

**FIGURE 9:**
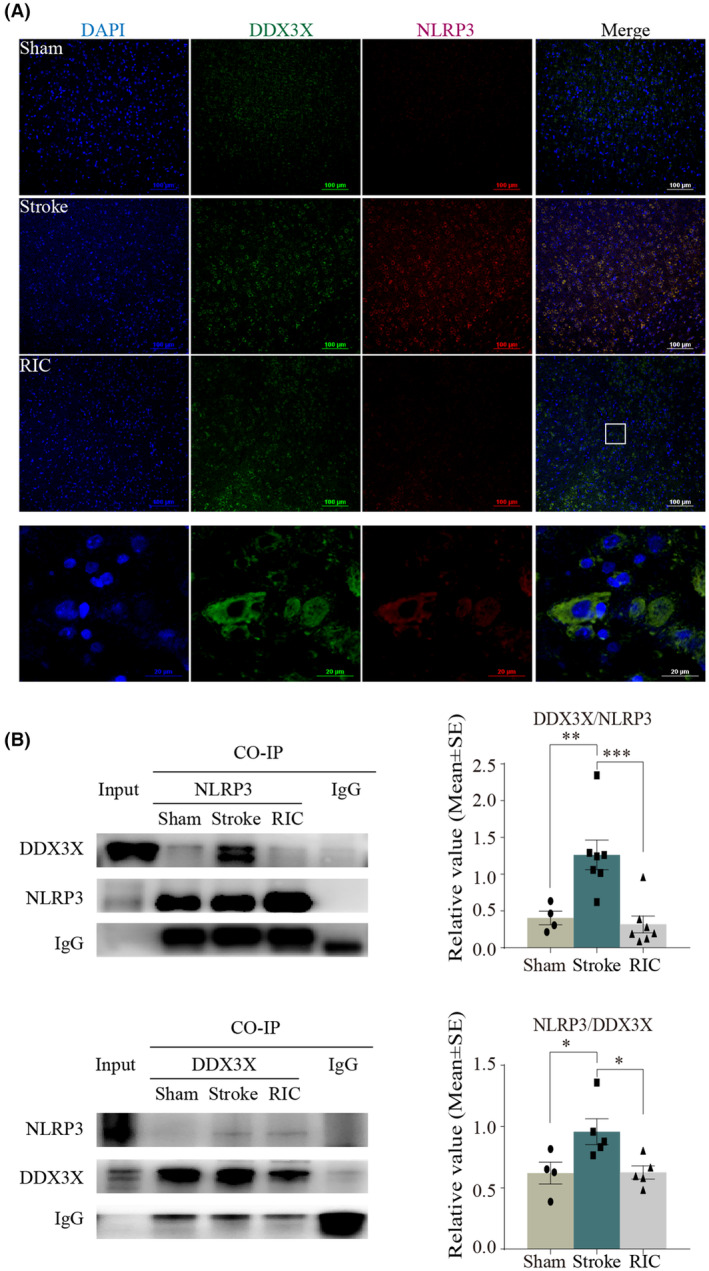
DDX3X interacted with NLRP3 in the MCAO model. (A) Representative merged images showing the colocalization of NLRP3 with DDX3X in brain tissue sections. Scale bar = 100 and 20 μm (green represented DDX3X; red represented NLRP3; yellow represented colocalization). (B) The co‐IP assay was used to detect the interaction between NLRP3 with DDX3X in the MCAO model. The data were presented as the mean ± SEM. *p < 0.05, **p < 0.01, ***p < 0.001.

We performed an immunofluorescence assay on the cerebral cortex with confocal laser microscopy to observe the interaction of DDX3X with SGs formation. The results showed that DDX3X was co‐localized with G3BP1 in the cortex and striatum (Figure 8A; yellow represented colocalization). To confirm the interaction between DDX3X and G3BP1, we performed a co‐IP assay and found that the binding effect between DDX3X and G3BP1 was significantly weakened after ischemia/reperfusion (*p <* 0.05), while early RIC enhanced the expression (*p <* 0.05, Figure 8B). These data suggested DDX3X was an essential component of SGs in the MCAO model, and RIC recruited DDX3X to SGs and stimulated its formation. To observe the interaction of DDX3X with NLRP3 inflammasome, we also performed the immunofluorescence assay on the cerebral cortex with confocal laser microscopy and co‐IP assay in brain tissues. The results showed the physical interaction between NLRP3 and DDX3X was remarkably augmented in ischemic rats (*p <* 0.05), which was attenuated by RIC (Figure 9A, yellow represented colocalization and *p <* 0.05, Figure 9B). The data suggested that DDX3X was critical for the NLRP3 inflammasome activation in the MCAO model and that RIC recruited DDX3X to SGs to compromise NLRP3 inflammasome activation. It is likely that the stress granule protein DDX3X interacted with NLRP3 to drive inflammasome activation, while RIC inhibited NLRP3 inflammasome activation via stimulating SGs to sequestrate DDX3X.

## 4 DISCUSSION

In the present study, after cerebral ischemia/reperfusion, we combined early RIC with subsequent exercise at post‐stroke Day 5 to establish a new rehabilitation strategy. RIC followed by exercise was better able to improve long‐term functional outcomes with reduction of brain injury and increased neuroplasticity, as compared to monotherapy with RIC or exercise alone. We further demonstrated that the neuroprotection by early RIC was due to SGs formation and sequestrated DDX3X molecules, which in turn led to the inhibition of NLRP3 inflammasome within 3 days.

The pathophysiological changes that occur after stroke are a dynamic process,^7^ which provides a basis for the selection of the RIC and exercise scheme adopted in our new strategy. In the acute stage (within 24 h post‐stroke), tissue around the infarction is less damaged and begins to recovery spontaneously. In the subacute phase (1 to 5 days post‐stroke), spontaneous recover in the penumbra reaches its peak. In the chronic phase (5 days to several months post‐stroke), endogenous repair‐related events in the brain are relatively stable, and brain structure and function could not be modified unless there is a specific intervention. Neurons around the infarction are vulnerable and sensitive to injury in the acute stage after stroke.^7^, ^8^ Exercise initiated within the acute stage actually exacerbates brain injury,^10^ but RIC exerts neuroprotective effects in the post‐stroke brain of the acute period, safely and feasibly.^18^, ^43^ Therefore, our current study initiated RIC at hour 6 after reperfusion to establish an optimal biochemical and physiological setting. The role of exercise in stroke rehabilitation cannot be understated. Besides inducing nerve remodeling, exercise can significantly improve the balance and coordination ability of stroke patients. Exercise also plays an indispensable role in the psychological, spiritual, emotional, and other aspects of patients as it enables them to directly participate and to observe measurable changes in their rehabilitative progress.^3^, ^23^, ^24^ Therefore, it is worthwhile to discern treatment regimens that are complimentary to the exercise‐based rehabilitation treatment framework. The previous studies also proposed that exercise rehabilitation was mostly beneficial when started in the period during which the processes of neuroplasticity reach homeostasis and have long‐term recovery potential after stroke.^6^, ^44^ Multiple studies have suggested that exercise or RIC solely could assuage cerebral harm and improve functional outcomes after ischemic stroke.^14^, ^15^, ^16^, ^20^ Our data particularly indicated that RIC followed by exercise mitigated brain injury and provided an additive benefit on long‐term sensory, motor, and cognitive improvement after stroke. Notably, our novel approach was found to be more effective in terms of functional recuperation than either RIC or exercise alone. Furthermore, the present study provided comparable evidence of enhanced synaptogenesis (SYN, PSD‐95, and BDNF) and angiogenesis (VEGF, Ang‐1, and Ang‐2). Many studies have indicated that these proteins contributed to long‐term sensory motor and cognitive function in the brain.^45^, ^46^, ^47^, ^48^ RIC followed by exercise maximized the therapeutic potential of each alone by minimizing occurrence of adverse events that may harm vulnerable neurons during the acute phase,^7^, ^8^ and consolidating neuroplasticity in the subacute phase of stroke.^7^

**FIGURE 10:**
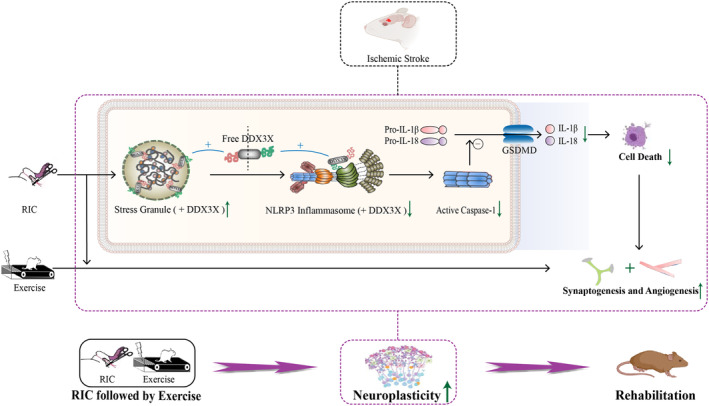
Schematic of RIC followed by exercise mechanisms in which RIC was followed by exercise‐induced rehabilitation. RIC followed by exercise‐promoted rehabilitation in ischemic rats by enhancing neuroplasticity. First, RIC increased SGs formation at an early stage within 3 days, which, in turn, consumed DDX3X and then inhibited the NLRP3 inflammasome, which mediates neuronal cell death. Thus, early RIC set the stage to optimize the environment for efficient synaptogenesis and angiogenesis. In addition, RIC followed by exercise‐enhanced synaptogenesis and angiogenesis, for better long‐term neurorehabilitation.

To establish new rehabilitation strategies with a solid theoretical foundation, it is necessary to delve deeper into the molecular regulatory mechanisms. Previous studies indicate that NLRP3 is activated by ischemia/reperfusion stimuli, which leads to the recruitment of the adapter ASC, and then recruits the procaspase‐1 resulting in its cleavage and activation, inducing the maturation and secretion of IL‐1β and IL‐18, leading to neuronal cell death and aggravating brain injury.^29^, ^30^ It is well established that RIC is associated with reduced levels of the pro‐inflammatory cytokines following cerebral infarction.^37^, ^49^, ^50^ In intestinal I/R injury mouse models, RIC was demonstrated to suppress the NF‐κB/NLRP3 inflammatory pathways via the ERK pathway.^51^ A link between early RIC after ischemic stroke with NLRP3 inflammasome was suggested in our current study, and furthermore, investigations are warranted to examine a cause‐and‐effect relationship. Induction of SGs has been proved to compromise NLRP3 inflammasome activation.^33^ SGs are associated with the self‐protection mechanism, which is assembled in the cytoplasm when cells are stressed.^33^ The assembly and disassembly of SGs are dynamic processes under strict regulation.^33^ In line with this, our study found that expressions of G3BP1, TIA1, and DDX3X^33^, ^52^ and the major components of SGs were transiently increased at 0 h after cerebral ischemia/reperfusion, and then decreased gradually until 24 h postreperfusion. This suggested the dynamic generation of SGs at different time points of reperfusion. In the acute stage of stroke, ischemia/reperfusion injury stress may stimulate SGs formation to promote the self‐protection of neurons, but SGs gradually deaggregate after reperfusion and the self‐protection of neurons is weakened. A recent study showed that miR‐335 promoted SG formation in acute ischemic stroke, which provided a possible therapeutic target for brain injury.^53^ The present results suggested that early RIC may stimulate SGs generation, leading to neuroprotection. Furthermore, investigation is warranted to determine SG formation on NLRP3 inflammasome activation in response to early RIC after stroke. DDX3X, an essential component of SGs, has been shown to be critical for the NLRP3 inflammasome activation. In an in vitro model of cerebral ischemia‐reperfusion injury, the knockdown of DDX3X attenuated NLRP3 inflammasome activation and pyroptosis induced by oxygen‐glucose deprivation/reoxygenation (OGD/R).^54^ The assembly of SGs could consume DDX3X and then inhibit NLRP3 inflammasome activation, and reduce cell death.^33^ In the present study, RIC was an ischemic/hypoxia stress that promoted the expressions of G3BP1, TIA1, and DDX3X, and enhanced the interaction between DDX3X and G3BP1. Moreover, RIC significantly inhibited the NLRP3 inflammasome activation and weakened the interaction between DDX3X and NLRP3. Based on these studies, it is likely that RIC alleviated brain damage via stress‐granule‐mediated inhibition of the NLRP3 inflammasome, and thus mitigated brain injury, which, in turn, reduced the burden of neuronal repair and promoted efficient synaptogenesis and angiogenesis for a better neurorehabilitation (Figure 10). The data suggest that SGs, DDX3X, and NLRP3 inflammasome could potentially serve as therapeutic targets for the treatment of ischemic stroke.

## 5 PERSPECTIVES AND PROSPECTIVE

Our study presents a transformative stroke rehabilitation approach, where remote ischemic conditioning (RIC) is followed by exercise, demonstrating a superior rehabilitation impact on ischemic rats compared to either RIC or exercise alone. The effectiveness of this novel strategy is primarily underpinned by the early neuroprotective effect of RIC, which alleviates ischemia‐reperfusion injury via stress‐granule‐mediated inhibition of the NLRP3 inflammasome. The strength of our research was twofold. Initially, we aimed to discern whether early RIC could mitigate ischemia‐reperfusion injury within the first 3 days, while elucidating the molecular mechanisms driving this process. The reduction in brain injury was quantified by measuring the infarct volume, verifying that RIC indeed delivered a neuroprotective effect. Simultaneously, our study sought to understand how our innovative rehabilitation protocol could improve long‐term functional outcomes. By employing RIC followed by exercise, we observed enhanced long‐term functional recovery as opposed to focusing on short‐term brain infarction alone.

In conclusion, our study affirmed the importance of early RIC in comprehensive rehabilitation by ameliorating ischemia‐reperfusion injury via the inhibition of the NLRP3 inflammasome by stress granules (SGs). Therefore, monitoring the dynamic pathological changes following stroke can shed light on the scientific significance and the effective mechanisms of our novel rehabilitation strategy. Moreover, stress granules, DDX3X, and the NLRP3 inflammasome emerged as promising therapeutic targets for ischemic stroke treatment. Future studies should delve into exploring the dynamic multi‐target regulation mechanisms that amalgamate neuroprotective and neuroplasticity strategies for optimal rehabilitation.

## AUTHOR CONTRIBUTIONS

Qingzhu Wang, Xiaokun Geng, and Yuchuan Ding designed the study, conducted the experiments, collected and analyzed the data, and drafted the manuscript. Wesley Kohls, Melissa Wills, Fengwu Li, and Qi Pang helped interpreting the data and made critical revision to the manuscript. All authors have read and agreed to the published version of the manuscript.

## FUNDING INFORMATION

This work was partially supported by the National Natural Science Foundation of China (81871838, 82002382), the Science and Cultivation Fund of Capital Medical University (PYZ21170), the Tongzhou District Health Development Research Project (KJ2023CX026), the Beijing Tongzhou District Financial Fund (2023), and the Laboratory Development Funds of Luhe Hospital (2022).

## CONFLICT OF INTEREST STATEMENT

The authors declare that they have no conflict of interest.

## Supporting information


Appendix S1.

## Data Availability

Data and materials related to the current study can be accessed from our corresponding author upon reasonable request.
