# Supplementary material for: A novel stroke rehabilitation strategy and underlying stress granule regulations through inhibition of NLRP3 inflammasome activation
Source: CNS Neurosci Ther. 2023 Aug 15;30(1):e14405. doi: 10.1111/cns.14405 (PMC10805392; doi:10.1111/cns.14405)

Full unedited blot for Figure 4B

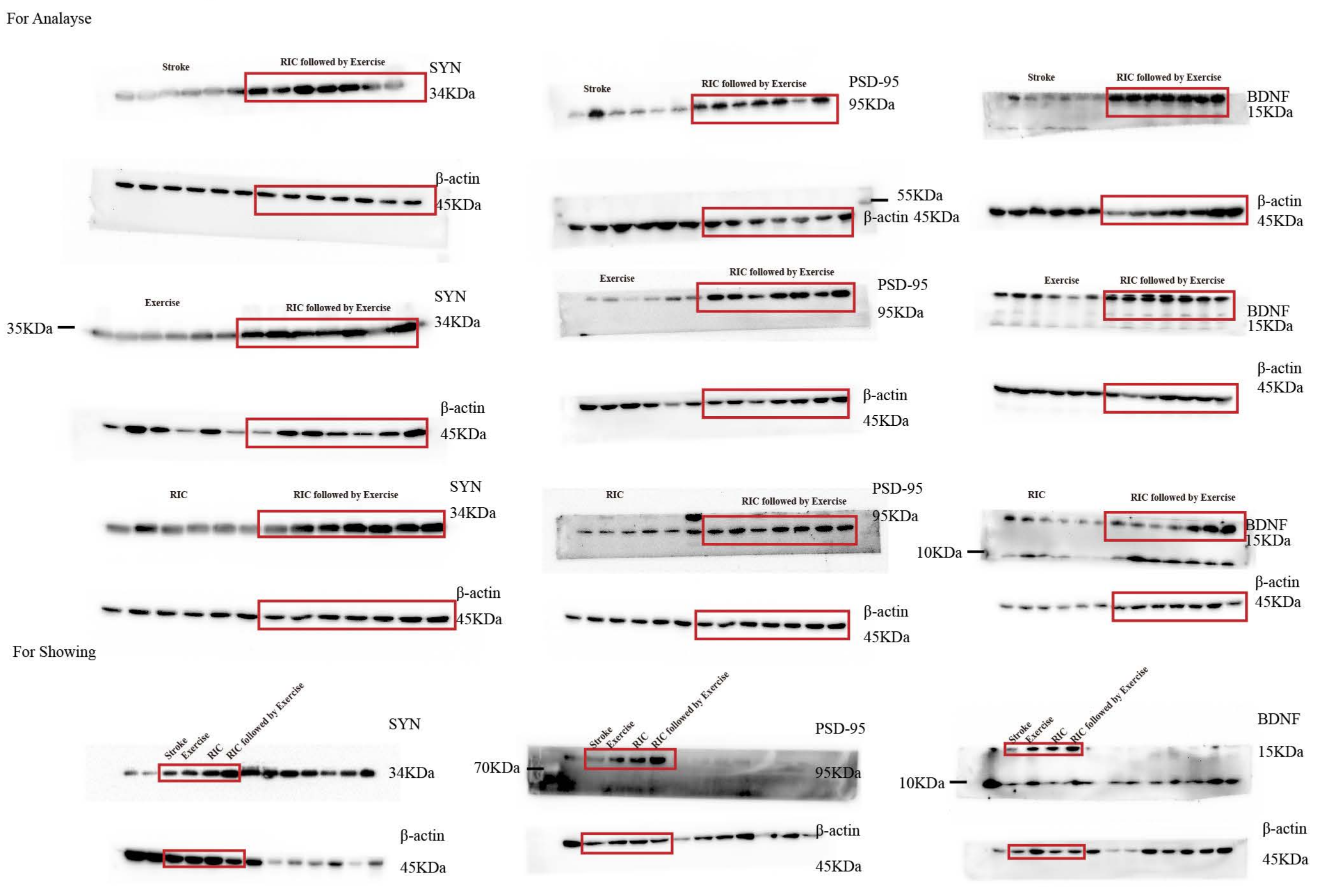

Full unedited blot for Figure 5B

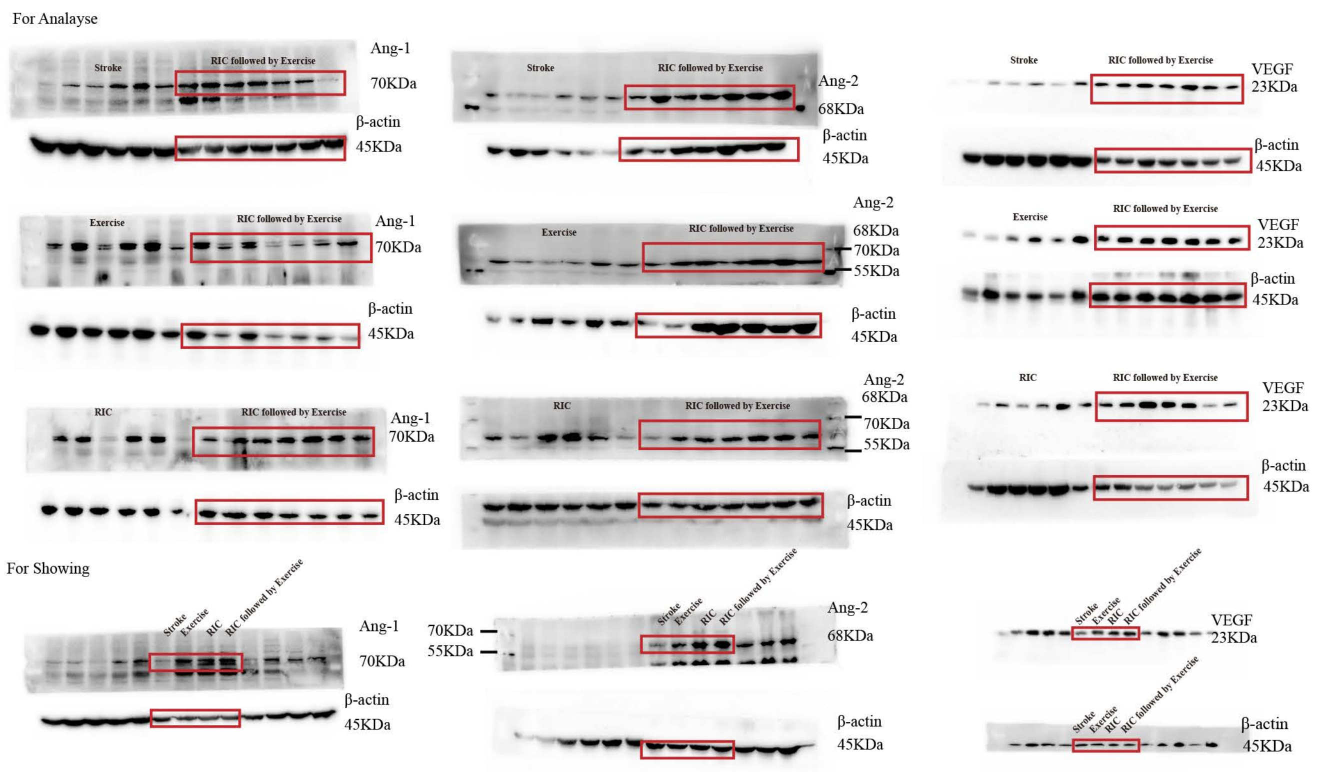

Full unedited blot for Figure 6C

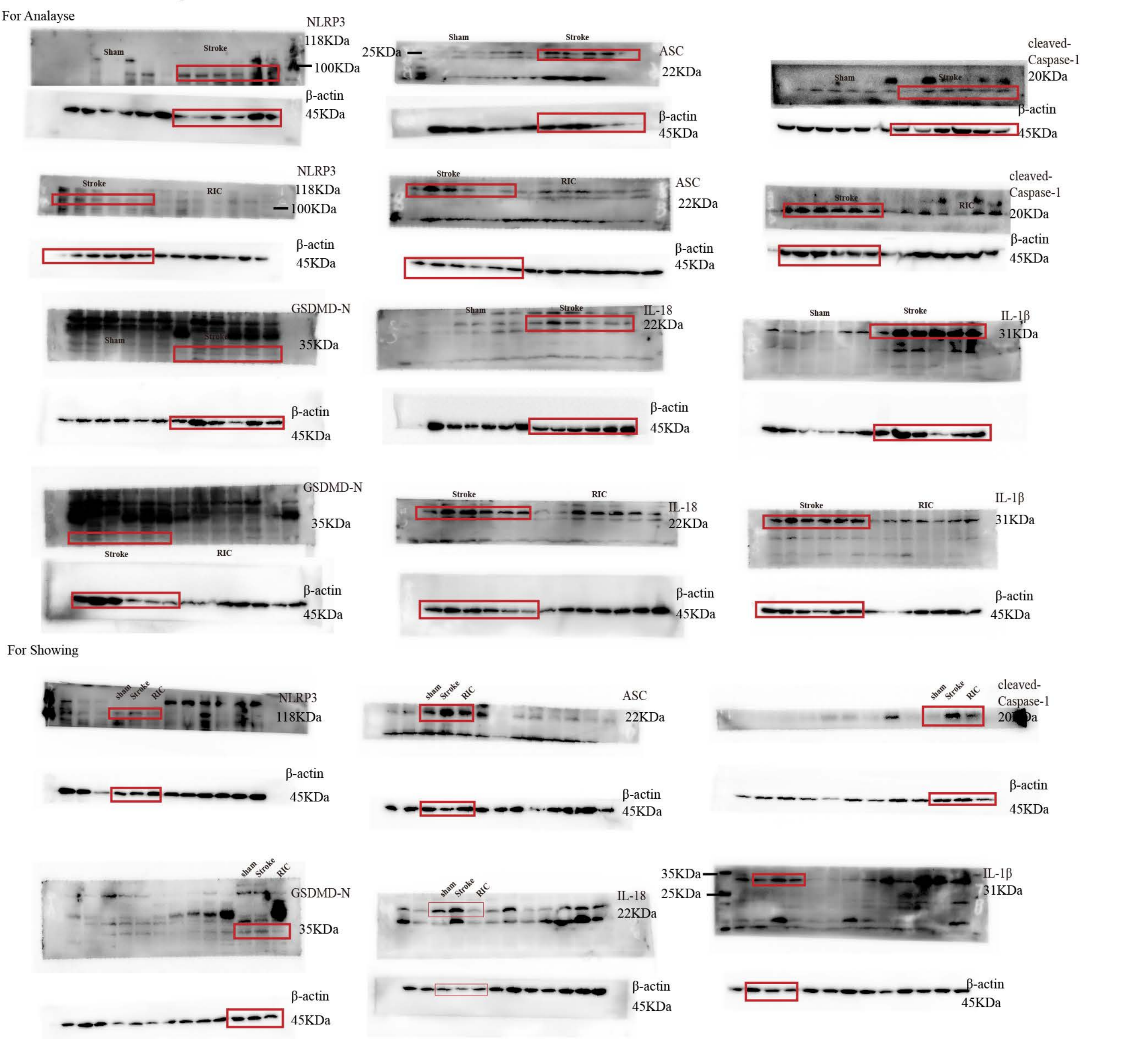

Full unedited blot for Figure 7B

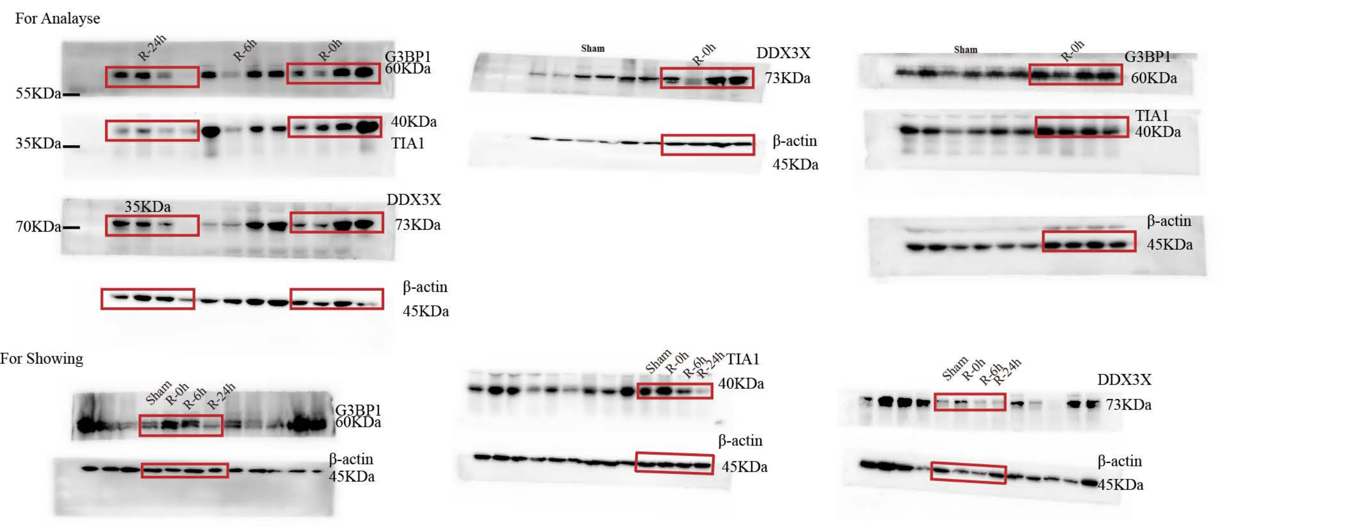

Full unedited blot for Figure 7D

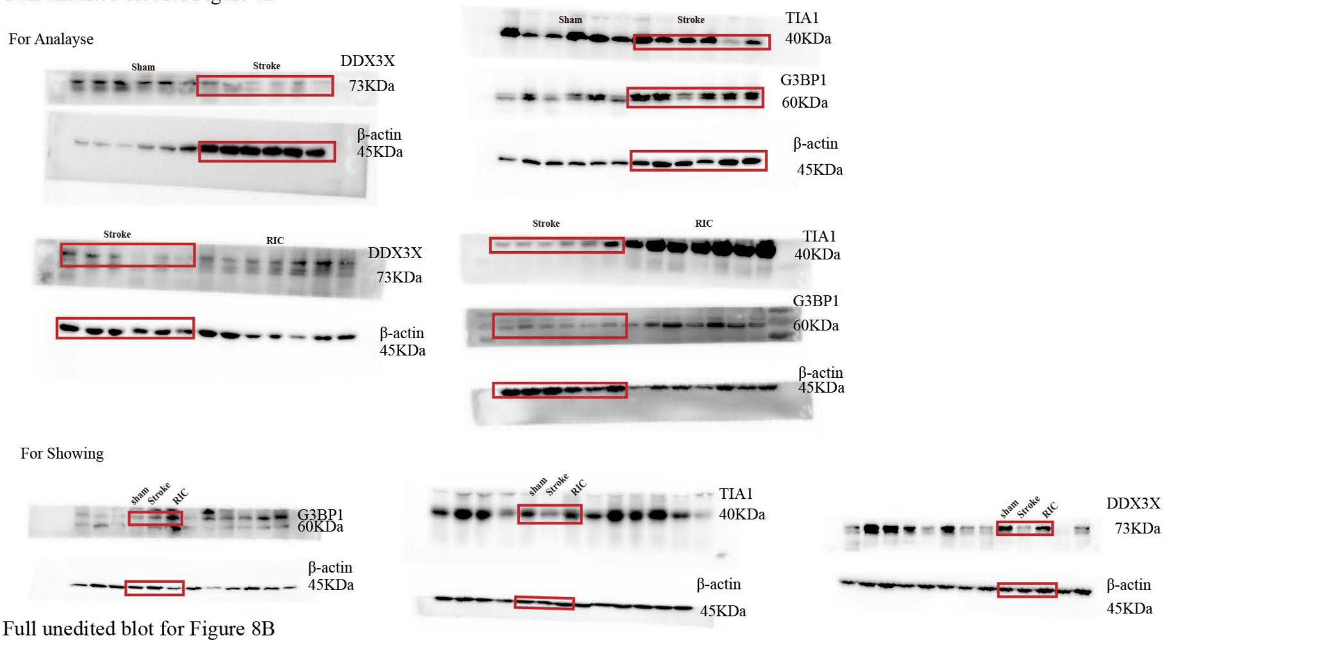

Full unedited blot for Figure 8B

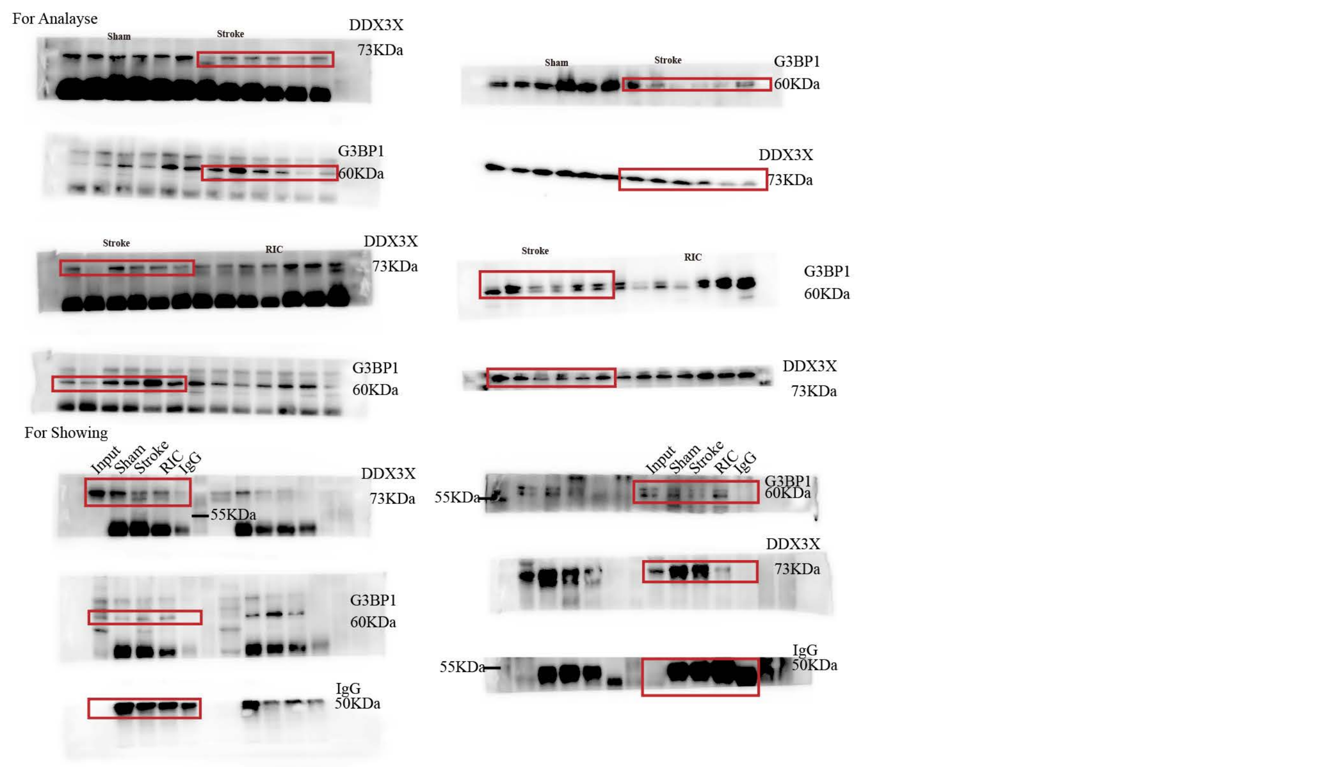

Full unedited blot for Figure 9B

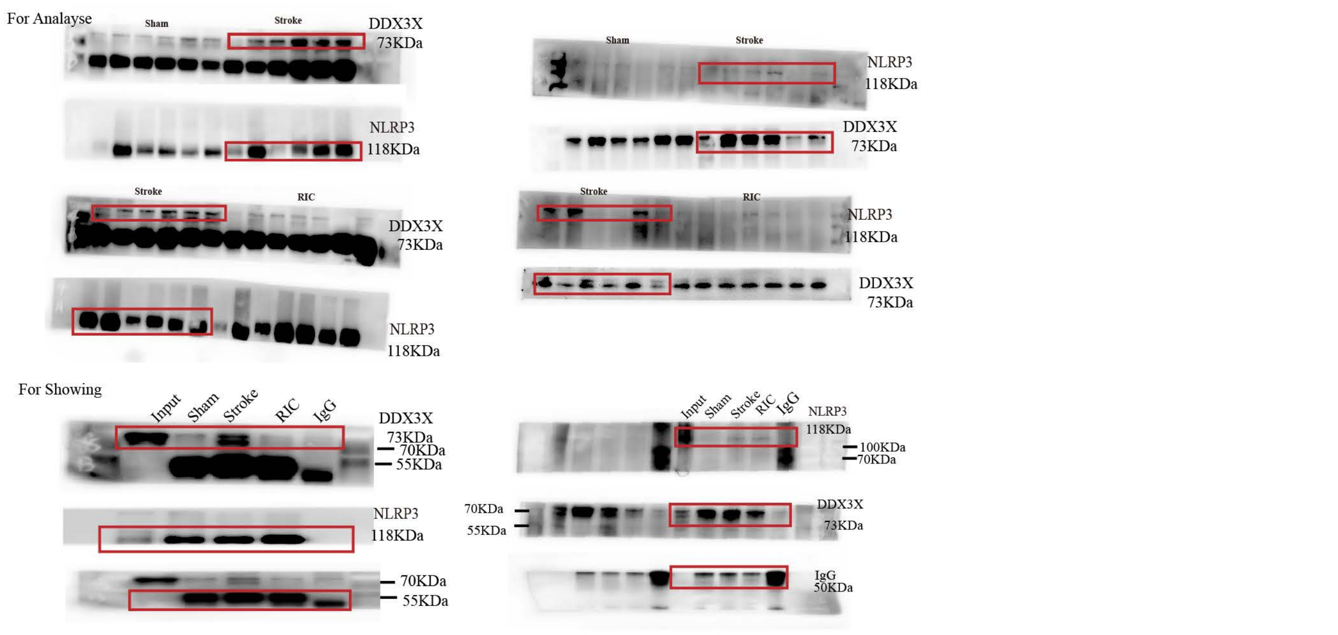

Supplement: Supplementary file 1 — Appendix S1. [file CNS-30-e14405-s001.pdf]
